# Applications of Aptasensors in Clinical Diagnostics

**DOI:** 10.3390/s120201181

**Published:** 2012-01-30

**Authors:** Ping Hong, Wenli Li, Jinming Li

**Affiliations:** 1 National Center for Clinical Laboratories, Beijing Hospital, Beijing 100730, China; E-Mails: pinghong98@yahoo.com (P.H.); totolwl@163.com (W.L.); 2 Graduate School, Peking Union Medical College, Chinese Academy of Medical Sciences, Beijing 100730, China

**Keywords:** biosensor, aptamer, aptasensors, clinical diagnostics

## Abstract

Aptamers are artificial oligonucleotides (DNA or RNA) selected *in vitro* that bind a broad range of targets with high affinity and specificity; a sensitive yet simple method to utilize aptamers as recognition elements for the development of biosensors (aptasensors) is to transduce the signal electrochemically. So far, aptasensors have been applied to clinical diagnostics and several technologies are in development. Aptasensors will extend the limits of current clinical diagnostics. Although the potential diagnostic applications are unlimited, the most current applications are foreseen in the areas of biomarker detection, cancer clinical testing, detection of infectious microorganisms and viruses. This review attempts to list examples of the research progresses of aptamers in biosensor platforms that have been published in recent years; in particular, we display cases of aptasensors that are already incorporated in clinical diagnostics or have potential applications in clinical diagnostics.

## 1. Introduction

High requirements regarding human health have resulted in an increasing number of clinical tests, so there is a rising need to develop more sensitive, reliable, time-efficient and inexpensive methods of analysis. Conventional techniques, such as molecular assays (immunological or nucleic acid technologies) and microbial culture-based tests, are either time consuming or require sophisticated equipment and expensive. Biosensor technology is probably one of the fastest growing areas to solve some of the problems concerning sensitive, rapid and cheap measurement [1].

A biosensor is defined as a compact analytical device incorporating a molecular recognition entity associated with or integrated with a physicochemical transducer [2]. It consists of two main components, a biological component, which reacts with a target substance, and a signal-generating component, which detects the resulting products. More specifically, we can consider a biosensor as a type of biomolecular probe that weighs the presence or concentration of biological molecules or biological structures by translating a biochemical interaction at the probe surface into a quantifiable physical signal. Biosensors are already used for several clinical applications, such as for electrochemical measurement of blood glucose concentrations. The first generation of biosensors were catalytic systems that integrated particularly enzymes with transducers that transferred the biological reaction into an electronic signal. The next generation of biosensors, affinity biosensors, utilized different biological elements, such as antibodies, receptors (natural or synthetic). Furthermore, the reaction between the target and the immobilized biomolecule on the transduction element is controlled by affinity interactions, such as the antigen-antibody, the DNA-DNA.

Recognition molecules are the key element of biosensors since the sensitivity and specificity of the sensing elements will play an important role in the success of the sensor device [3]. A range of recognition molecules have been employed for clinical diagnosis. However, the use of antibodies in biosensor detection methods and in the analysis of very complex samples could partially be hampered by the nature and synthesis of these protein receptors. In order to circumvent some of these drawbacks, other recognition molecules, such as aptamers, are being explored as alternative. This review attempts to list examples of the research progress of aptamers in biosensor platforms that have been published in recent years; in particular, we display cases of aptasensors that are already incorporated in clinical diagnostics or have potential applications in clinical diagnostics.

## 2. Aptasensors

### 2.1. Aptamers and Biosensors

Aptamers (derived from the latin *aptus*, meaning to fit) are artificial nucleic acid (DNA or RNA) ligands which can be selected from combinatorial libraries of synthetic nucleic acids, possessing specific binding characteristics to their targets. Using the systematic evolution of ligands (SELEX) process, aptamers can be isolated from randomly synthesized RNA or DNA pools and produced. Numerous high-affinity and highly specific aptamers have been generated against a wide variety of target molecules including small organics, peptides, proteins, and even complex-target such as whole cell [4,5]. In addtion, aptamers can be selected against non-immunogenic and toxic targets, which make the technique superior to antibodies, enabling the design and synthesis of probes or capture molecules for such targets.

Biosensors that employ aptamers as a recognition element are called aptasensors [6]. DNA and RNA aptamers can be modified chemically to undergo analyte-dependent conformational changes. For instance, Aptamers can be modified for immobilization purposes and incorporated particular reporters, without influencing their affinity, which has aided varieties of design methods [7–12]. Aptamers can also be engineered to withstand repeated cycles of denaturation and renaturation; this opens up a possibility to regenerate the immobilized biocomponent function for reuse [6,13]. In addition, they can be easily labeled for their use in diagnostics [14].

### 2.2. Signal-Transduction

Development of aptasensors has been carried out with various detection schemes, from label-free methods, such as surface plasmon resonance (SPR) [15], and quartz crystal microbalance (QCM) measurements, to other methods often requiring labels, such as electrochemistry[16], fluorescence [17–19], chemiluminescence [20], field effect transistors [10,16], which have been reported and enhanced the advancement of this field. Alternatively, sensors often utilize labeled target molecules or secondary capture agents (in the case of a sandwich-type assay) which themselves contain some forms of label or reporter molecule. So far, there are mainly two types of aptasensors to apply, including electrochemical and optical aptasensors.

#### 2.2.1. Electrochemical Aptasensors

A typical electrochemical aptasensor makes use of an electrode surface as the platform to immobilize biological sensing aptamer, for which the analyte-binding event is monitored based on electrochemical current variations. Electrochemical transduction offers the advantages of high sensitivity, which can be enhanced by attaching biocatalytic labels to the aptamer-target complexes to amplify the detection signal, readily miniaturized, and low cost of production since they do not require expensive optical instruments [16,21]. Additionally, it is possible to use label-free and reusable detection systems.

#### 2.2.2. Optical Aptasensors

Optical aptasensors include aptamers labeled with fluorescence, luminophore, enzyme, nanoparticles) or aptamer with label-free detection systems (e.g., SPR, optical resonance) [22]. Most methods are mainly based on a labeling approach. Some targets can be optically detected using not only colorimetry, chemiluminescence or the most developed fluorescence mode but also more recent non-conventional optical methods such as surface plasmon-coupled directional emission (SPCDE) [22].

Different label-free techniques have recently been shown to be suitable for developing aptasensors or aptamer-based microarrays, such as surface plasmon resonance (SPR), diffraction grating, evanescent-field-coupled (EFC) waveguide-mode, optical resonance or Brewster angle straddle interferometry (BASI) [22]. SPR biosensors prepared using optical fibers can be used as a cost-effective and relatively simple-to-implement alternative to well established biosensor platforms for monitoring biomolecular interactions *in situ* or possibly *in vivo* [23].

## 3. Application of Aptasensors in Clinical Diagnosis

Beyond all these features, the applications of aptamers as bio-compotents have offered extraordinary prospects in diagnostic assays [24]. Here we briefly describe a few examples of the use of aptasensors in the areas of biomarker detection, cancer clinical testing, and detection of infectious microorganisms.

### 3.1. Application of Aptasensors for Detection of Biomarkers

The analysis of biomarkers in blood, urine and other body fluids is one of the methods applied in the early detection of diseases. Aptasensors have been used for detection of biomarkers, such as thrombin, Immunoglobulin (Ig) E. A list of examples of the available reports of aptasensors for different biomarkers is provided in Table 1. Furthermore, representative examples of aptasensors for detection of thrombin are discussed in the following section.

Thromin is an enzymatic active, circulating biomarker for blood coagulation activity levels. Clinical studies, applying assays and strategies applicable for endogenous plasma thrombin measurements, are needed to evaluate the clinical impact of thrombin. Nowadays, many assays, based on the thrombin-binding aptamer for the detection of thrombin, have been developed in recent years. Mostly, thrombin has been used as a model for detection in the aptasensors assays. The below are three cases.

**Scheme 1.**
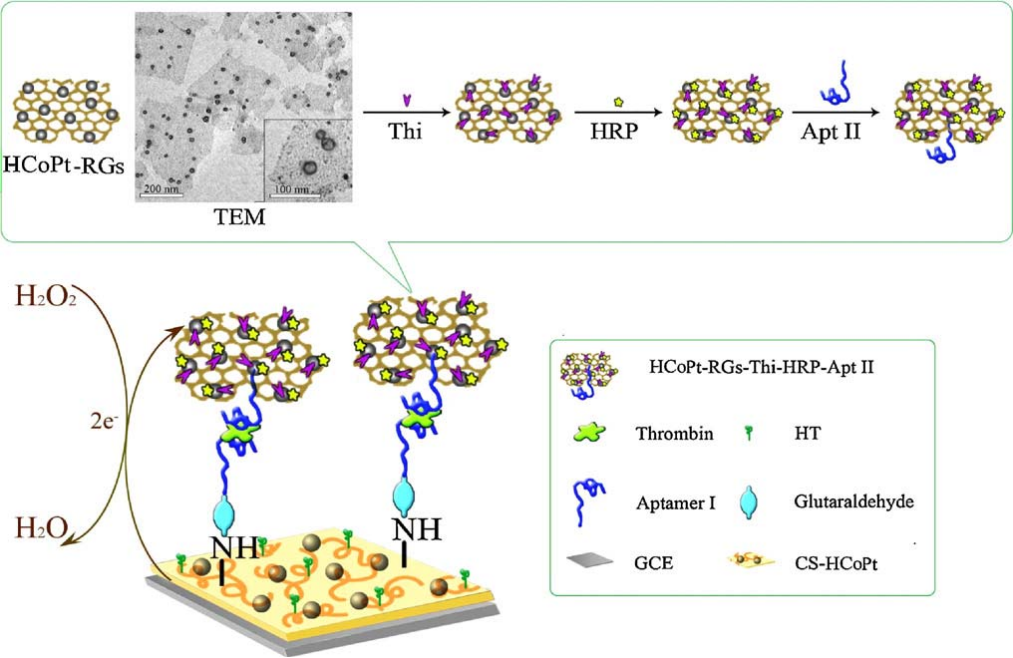
Schematic diagrams of preparation of the sandwich-type electrochemical aptasensor and TEM images of the hollow CoPt alloy nanoparticles decorated graphene. Reproduced with permission form Reference [25] (Abbreviations: HT, hexanethiol; GCE, glassy carbon electrode; CS-HCoPt, the chitosan-hollow CoPt alloy nanoparticle).

An advanced sandwich-type electrochemical aptasensor assay coupled to use the hollow CoPt alloy nanoparticle (HCoPt)-RGs conjugates as secondary label demonstrated a good sensitivity and selectivity, with a limit of detection (LOD) of 3.4 × 10^−13^ M. Scheme 1 illustrates the fabrication of electrochemical strategy with the aptamer-linked sandwich assay for the ultrasensitive detection of thrombin. The formed conjugates provided large surface area for loading plentiful redox probe thionine (Thi), horseradish peroxidase (HRP) and secondary aptamer (Apt II) with good stability and friendly biocompatibility [25].

Also, a sandwich electrochemical aptasensor assay was developed for thrombin based on amplification of aptamer-gold nanoparticles-horseradish peroxidase (aptamer-AuNPs-HRP) conjugates [26]. In this electrochemical protocol, aptamer1 (Apt1) was immobilized on core/shell Fe_3_O_4_/Au magnetic nanoparticles (AuMNPs) and served as capture probe. Aptamer2 (Apt2) was dual labeled with AuNPs and HRP and used as detection probe. In the presence of thrombin, the sandwich format of AuMNPs-Apt1/thrombin/Apt2-AuNPs-HRP was fabricated. Remarkable signal amplification was realized by taking the advantage of AuNPs and catalytic reactions of HRP. Other proteins, such as human serum albumin, lysozyme, fibrinogen, and IgG did not show significant interference with the assay for thrombin. Linear response to thrombin concentration in the range of 0.1–60 pM and the LOD of 30 fM (S/N = 3) was obtained with the proposed method. This electrochemical aptasensor was simple, rapid (the whole detection period for a thrombin sample is less than 35 min), sensitive and highly specific, it showed promising potential in protein detection and disease diagnosis [26].

Meanwhile, aptasensor assays can be used for detection with various working principles. Pu *et al.* [27] have displayed a smart polymeric transducer and aptamer/intercalating dye system that allows the label-free detection of thrombin with high sensitivity and selectivity. Thrombin detection was completed through the simple “mix and detect” mode. In addition, the methodology didn’t need any chemical modification on the probes or the analytes. The amount of the thrombin could be measured by the fluorescence intensity changes. The LOD was estimated to be 0.1 nM. This strategy might offer a new strategy to detect a wide spectrum of analytes and would be highly beneficial in future clinical applications.

### 3.2. Application of Aptasensors for Cancer Clinical Testing

With the growing number of cancer cases being diagnosed worldwide, cancer is the second most common cause of mortality and morbidity in the World due to late disease detection. Early diagnosis is crucial in all cancers to improve patient survival and disease prognosis, and may lead to cancer prevention- and for this reason sensitive and specific methods are required for early cancer diagnosis.

Detection and identification of tumor cells rely on the identification of some markers that appear only on the tumor cells such as lymphoma (Ramos) cells, leukemia cells. Identification of these cancer markers requires specific probes to bind in order to find the potential risk factor. In blood cells, plasma proteins or free DNA may be tumor markers. A number of recent studies have successfully used aptamers for targeting tumor markers.

**Scheme 2.**
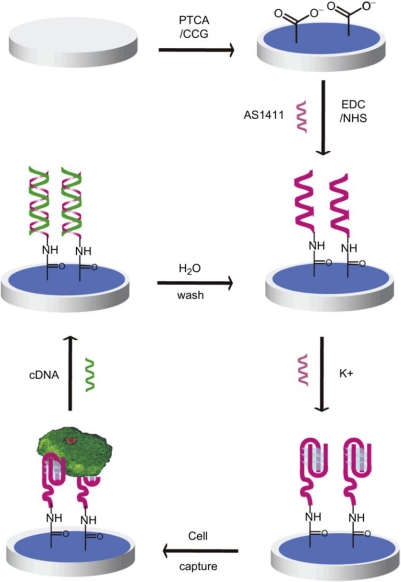
Schematic representation of the reusable aptamer/graphene-based aptasensor. The sensor is constructed based on graphene-modified electrode and the first clinical trials II used aptamer, AS1411. AS1411 and its complementary DNA are used as a nanoscale anchorage substrate to capture/release cells. Reproduced with permission form Reference [39]. (Abbreviations: CCG, chemical converted grapheme; EDC, 1-ethyl-3-(3-dimethylaminopropyl) carbodiimide; NHS, N-hydroxysuccinimide).

Feng *et al*. [39] reported an example for label-free cancer cell detection by using an electrochemical sensor based on the first clinical oncology trial II used aptamer AS1411 and graphene-modified electrode. The aptamer-PTCA (perylenetetracarboxylic acid) nanocomposite was utilized as nanoscale anchorage substrates to effectively capture cells on electrode surface through the specific binding between cell surface nucleolin and the aptamer AS1411 (Scheme 2). The electrochemical aptasensor can distinguish cancer cells and normal ones and detect as low as one thousand cells. With DNA hybridization technique, this E-DNA sensor can be regenerated and reusable for cancer cell detection.

Platelet-derived growth factor B chain (PDGF-BB) is a potential cancer marker and is known to be related to tumor growth and transformation. Recently, Chai *et al.* [40] developed a sensitive and low cost ECL aptasensor for PDGF-BB by assembling N-(aminobutyl)-N-ethylisoluminol functionalized gold nanoparticles (ABEI-AuNPs) with aptamers as nanoprobes. The biotinylated aptamer capture probes were first immobilized on a streptavidin coated gold nanoparticle (AuNPs) modified electrode, then, a sandwich conjugate modified electrode was carried out by successively attached the target PDGF-BB and the ABEI-AuNPs tagged aptamer signal probe. ECL measurement was run with a double-step potential in carbonate buffer solution containing H_2_O_2_, showing high sensitivity and specificity. The detection limit was as low as 2.7 × 10^−^^14^ M. In addition, the ABEI-AuNPs signal amplification was more simple, stable, practical, and sensitive compared to other reported signal amplification strategies based on aptamer-related polymerase chain reaction or functionalized nanoparticles. Moreover, 7 human serum samples were measured by using the proposed aptasensor. The obtained result were in good agreement those determined by the PDGF-BB ELISA as the reference method, which indicated that it was feasible to apply the proposed ECL aptasensor to detect PDGF-BB in real human serum samples. Table 2 lists examples of aptasensors for cancer clinical testing in recent years.

### 3.3. Aptasensors for Detection of Microorganisms and Viruses

Detection, identification and quantification of microbial pathogens are crucial for public health protection. Similarly, identification of virally infected cells is essential for the study of the mechanism of viral diagnosis. Virus-infected cells display certain viral proteins, which are detected using probes for the detection of infection. Up to date, the ability to design probes for specific viral targets on the host cell surface is very limited. Several aptasensors have been developed to detect microorganisms and viral proteins. One representative example of HIV-1 Tat aptamers developed for detection is discussed in the following part. In addition, Table 3 lists examples of aptasensors for detection of microorganisms and viruses.

Microgravimetric methods on piezoelectric quartz crystals are based on the change of the oscillation frequency of the crystal upon mass change at its surface owing to receptor-target binding (QCM). Then, this change of oscillation frequency is the signal that is detected. With this method, a label-free detection of the target is possible. Tombelli *et al*. [51] developed an aptasensor platform to detect HIV-1 Tat protein by immobilizing an RNA aptamer on a piezoelectric quartz crystal. The QCM-based aptasensor has also been compared with the corresponding SPR-based aptasensor. The two aptasensors were constructed using biotin–avidin linking onto the gold surface of the transducers (quartz crystals or chips) for the immobilization chemistry. Both platforms showed similar reproducibility, sensitivity and specificity. The linear range of SPR (1–2.5 ppm) was higher that of QCM (0–1.25 ppm) [51].

## 4. Conculsions

Aptamer science has now reached maturity, through its growing impact on biology and medicine. The therapeutic use of aptamers is now well established, as in December 2004 the first aptamer compound named Pegaptanib was approved for clinical use [54]. Conversely, the different fields of clinical diagnostics, when dealing with systems based on biomolecular interactions, are still under the supremacy of immunoassays but diagnostics studies are now showing that some of the limits of current clinical diagnostics can be circumvented by taking advantage of the differences between aptamers and antibodies, such as cost-effective synthesis, flexibility for signal transduction and detection [55]. The good news is that there are currently some more aptamers in clinical phase 2/3 trials, such as factor IXa (FIXa), Von Willebrand factor (Vwf). However, the potential values of those aptamers for clinical diagnosis have not been fully investigated. Up to now, there is still no good answers to the question why aptamers have not yet penetrated into the clinical laboratory [56] and only a few aptasensors for clinical diagnostics were described [56,57]. As long as more aptamers will be developed and specified, the more aptasensors promise to play an important role in the future development of diagnostic methods.

## Figures and Tables

**Table 1.** Examples of application of aptasensors for detection of biomarkers.

**Target biomarker**	**Aptamer**	**Detection type**	**Signal transduction**	**Linear range/LOD**	**Reference**
Thrombin	DNA labeled with the HCoPt-RGs conjugates	Sandwich-type	Electrochemical	1.0 × 10^−12^ to 5.0 × 10^−8^ M/3.4 × 10^−13^ M	Wang *et al.* (2011) [25]
Thrombin	DNA dual labeled with AuNPs and HRP	Sandwich-type	Electrochemical	0.1 to 60 pM/30 fM	Zhao *et al.* (2011) [26]
Thrombin	DNA	Label free detection	Fluorescence	0 to 0.02 μM/0.1 nM	Pu *et al.* (2009)[27]
Thrombin	DNA labeled with a hole injector, naphthalimide, and a flurophore, Alexa532	DNA charge transport	Fluorescence	5 pM to 5 nM/1.2 pM	Zhang *et al.* (2011) [28]
Thrombin	DNA SA-ALP and biotinylated labled	Sandwich-type	ECL	1 × 10^−15^ to 1 × 10^−8^ M/0.33 fM	Liao *et al.* (2011) [29]
IgE	DNA streptavidin labled	ELISA-like array	SPR	n.s./n.s.	Wang *et al*. (2008) [30]
IgE	DNA labeled with avidin monolayer	Direct detection	QCM chemiluminescence	2.5 to 200 μg L^−1^/2.5 μg L^−1^	Yao *et al.* (2009) [31]
IgE	DNA labeled with single PPy nanowire-based microfluidic	One step electrochemical deposition method	Electrochemical	0.1 to 100 nM/0.01 nM	Huang *et al.* (2011) [32]
IgE	DNA attached to carboxyl (COOH)-modified NCD surface	Direct and label-free detection	EIS	0.03 to 42.8 mug/mL/0.03 mug/mL	Tran *et al.* (2011) [33]
RBP4	single-stranded DNA	Label free detection	SPR	0.2 to 0.5 μg mL^−1^/75 nM	Lee *et al.* (2008) [34]
CRP	RNA biotinylated	Direct detection	Optical	n.s./0.005 ppm	Bini *et al.* (2008) [35]
CRP	DNA	Sandwich-type	Optical	10 microg/L to 100 mg/L/n.s.	Pultar *et al*. (2009) [36]
NT-proBNP	DNA with cocaine-binding	Sandwich-type	ECL	0.01 to 500 ng mL^−1^/0.77 pg mL^−1^	Mao *et al.* (2011) [37]
IFN gamma	DNA thiolated/MB redox tag	Direct detection	Electrochemical	10 nM/0.06 nM	Liu *et al.* (2011) [38]

Abbreviations: LOD, limit of detection; HCoPt-RGs, hollow CoPt alloy nanoparticle onto reduced graphene oxide sheet; AuNP, Au nanoparticles; HRP, horseradish peroxidase; IgE, Immunoglobulin E; QCM, quartz crystal microbalance; SPR, surface plasmon resonance; PPy, polypyrrole ; EIS, Electrochemical impedance spectroscopy; NCD, nanocrystalline; RBP4, retinol binding protein 4; CRP, C-reactive protein; NT-proBNP, N-terminal pro-brain natriuretic peptide; ECL, electrochemiluminescence; MB, methylene blue; IFN, interferon; n.s., not specified.

**Table 2.** Examples of application of aptasensors for cancer clinical testing.

**Cancer marker detected**	**Aptamer**	**Detection type**	**Signal transduction**	**Linear range/LOD**	**Reference**
HeLa cells, K562 cells, MDA-231 cells	DNA	Label free detection	Electrochemical	n.s./n.s.	Feng *et al.* (2011) [39]
PDGF-BB	DNA labeled with biotin	A sandwich conjugate modified electrode	ECL	1.0 × 10^−13^ to 1.0 × 10^−11^ M/2.7 × 10^−14^ M	Chai *et al.* (2011) [40]
Ramos cancer cell, CEM cells	DNA	ECL array with a novel cycle-amplifying technique	ECL	n.s./n.s.	Jie *et al.* (2011) [41]
Multi-marker or Ramos cells, CCRF-CEM cells, Toledo Cells	DNA-conjugated FRET NP	Simultaneous multiplexed analysis	fluorescence	n.s./n.s.	Chen *et al.* (2009) [42]
Ramos cancer cell	DNA	Label free detection	ECL	100 to 1,000 cells mL^−1^/58 cells mL^−1^	Hun *et al.* (2011) [43]
Leukemia cells	DNA conjugated apt-MBs	a magnet-quartz crystal microbalance system	QCM	1 × 10^4^ to 1.5 × 10^5^ cells mL^−1^/8 × 10^3^ cells mL^−1^	Pan *et al.* (2010) [44]
PSA	DNA labeled with FITC	Aptamer blotting assay	Chemiluminescence	40 to 100 nM/n.s.	Savory *et al.* 2010 [45]
MUC1	DNA labeled with single PPy nanowire-based microfluidic	One step electrochemical deposition method	Electrochemical	n.s./2.66 nM	Huang *et al.* (2011) [33]
MUC1	DNA labeled with QD	Aptamer-based detection with quantum-dot based fluorescence readout	Fluorescence	n.s./250 nM	Cheng *et al.* (2009) [46]
GSH	RNA	SPR analysis and isocratic affinity chromatography	SPR	n.s./n.s.	Bala *et al.* (2011) [47]
VEGF	RNA conjugated CPNTs	FET-type biosensor based on CPNTs-aptamer	Electrochemical	n.s./400 fM	Kwon *et al.* (2010) [48]

Abbreviations: PDGF-BB, platelet-derived growth factor B chain; PSA, prostate specific antigen; MUC1, Mucin 1; FRET NP, fluorescence resonance energy transfer nanoparticles; GSH, Glutathione; SPR, surface plasmon resonance; ECL, electrogenerated chemiluminescence; QD NCs, quantum dot nanoclusters; VEGF, Vascular Endothelial Growth Factor; CPNTs, carboxylated polypyrrole nanotubes; FET, field-effect transistor; QCM, quartz crystal microbalance; apt-MBs, conjugated magnetic beads; QD, quantum dot; n.s., not specified.

**Table 3.** Examples of application of aptasensors for detection of microorganisms and viruses.

**Microbial and viral targets**	**Aptamer**	**Detection type**	**Signal transduction**	**Linear range/LOD**	**Reference**
Bacillus thuringiensis	DNA	Aptamer-functionalized QDs	Fluorescence	n.s./1,000 CFU/mL	Ikanovic *et al.* (2007) [49]
*E. coli* DH5a	DNA	Aptamer-functionalized SWNT-FET arrays	Fluorescence	n.s./n.s.	So *et al.* (2008) [50]
HIV-1 Tat	RNA biotinylated	A QCM –based and an SPR-based biosensor	QCM SPR	QCM (0–1.2 ppm) SPR (0–2.5 ppm)/QCM (0.25 ppm)	Tombelli *et al.* (2005) [51]
HCV core antigen	RNA	Chip-based detection	Fluorescence	n.s./n.s.	Lee *et al.* (2007) [52]
Prion	RNA	3-dimensional analysis	Fluorescence	n.s./n.s.	Mashima *et al.* (2009) [53]

Abbreviations: QD, quantum dots; CFU, colony forming units; SWNT-FET, single-walled carbon nanotube field-effect transistor; QCM, quartz crystal microbalance; SPR, surface plasmon resonance; n.s., not specified.
